# Toward Personalized Rotator Cuff Physical Therapy Dosage Using a Machine Learning-Based Pilot Study with EMG

**DOI:** 10.3390/bioengineering13040483

**Published:** 2026-04-21

**Authors:** AmirHossein MajidiRad, Iram Azam, Japp Adhikari, Mehrnoosh Damircheli

**Affiliations:** 1 Department of Engineering Technology, Purdue University Northwest, Hammond, IN 46323, USA; 2 Mayo Clinic, Jacksonville, FL 32224, USA; 3 Department of Computer Information Technology, Purdue University Northwest, Hammond, IN 46323, USA; iazam@pnw.edu (I.A.); adhikarj@pnw.edu (J.A.); 4 Department of Mechanical Engineering, York College of Pennsylvania, York, PA 17403, USA; mdamircheli@ycp.edu

**Keywords:** rotator cuff injury, electromyography (EMG), kinematic analysis, machine learning in movement science, rehabilitation engineering

## Abstract

Rotator cuff injuries are among the most common musculoskeletal conditions that affect shoulder function and can ultimately impact quality of life. While physical therapy is essential in the care of rotator cuff injuries, the ideal dose of therapeutic exercises continues to be a significant clinical dilemma because of the generalized nature of rehabilitation protocols. This pilot study proposes a machine learning approach to personalize rehabilitation using surface electromyography (sEMG) data collected from eight healthy individuals by testing four key shoulder movements: scaption, internal rotation, external rotation, and external rotation at 90° abduction. In this research, the XGBoost algorithm was used to model muscle activation patterns by achieving a high predictive accuracy (*R*^2^ = 0.5325; MSE = 0.0084 μV^2^). Because sEMG reliably measures superficial muscle activity, a linear programming model was used to divide a 60 min therapy session in a way that increases activation of superficial muscles (such as deltoid and trapezius) while reducing strain on deep muscles (such as supraspinatus and infraspinatus). Three optimization scenarios were tested by reflecting a different clinical goal: prioritizing superficial muscles, minimizing deep muscle strain, or balancing both. Optimized time allocations assigned more time to external rotation at 90° abduction and scaption. This research demonstrates the potential for data-driven methods to transform rotator cuff rehabilitation through personalized and evidence-based treatment plans. The results enhance clinical practice by enabling adaptive rehabilitation planning and show that machine learning can support decision-making in complex muscle activation analysis with strong performance and low latency.

## 1. Introduction

The shoulder has a wide range of motion and is frequently used in daily activities. Because of this, it is especially prone to injury, with rotator cuff tears being one of the most commonly diagnosed conditions in orthopedic practice. The rotator cuff consists of four muscles: supraspinatus, infraspinatus, teres minor, and subscapularis. These muscles help keep the shoulder joint stable and allow it to move in many directions [1]. These deep muscles organize active and passive shoulder movements. Superficial muscles such as the deltoid and trapezius provide secondary support which can contribute to functional movement patterns [2,3]. Rotator cuff injuries are mainly the result of repetitive use or trauma in the muscles which leads to pain and functional issues, mainly among athletes and older adults [4,5].

While rotator cuff pathology is traditionally described in biomechanical terms, rehabilitation outcomes are influenced not only by muscle activation but also by psychosocial factors such as pain perception, motivation, and treatment adherence. Recent research emphasizes multimodal, person-centered models that integrate physical and behavioral determinants of recovery [6].

Physical therapy (PT) still remains a key component of conservative treatment of rotator cuff injuries. It helps to restore mobility and strength along with muscular coordination [7]. However, there is a persistent challenge in PT, i.e., determining the optimal “dosage” of therapeutic exercise. It is essential to balance intensity, duration, and type of movement to encourage healing without overloading any tissues [8,9]. Rehabilitation procedures are normally generalized or guided by clinician experience, which may not be patient-specific variables like muscular imbalances or functional goals [10]. Recent studies focus on rehabilitation programs to individual patients but data-driven personalization strategies still remain limited [11,12,13]

Rehabilitation dosing involves coordinated manipulation of neuromuscular load parameters that drive tissue adaptation and recovery [14]. In rotator cuff rehabilitation, protocols emphasize progressive loading, restoration of motor control, and selective activation of stabilizing muscles such as the infraspinatus and teres minor [15]. EMG-based evaluation has been widely used to assess the effectiveness of shoulder rehabilitation exercises and to guide clinical decision-making in this population [16].

Surface EMG analysis typically requires preprocessing to extract reliable activation measures from raw signals. Sliding-window segmentation and RMS amplitude estimation are widely accepted techniques for quantifying muscle activation and reducing noise in dynamic movement analysis [17,18,19]. These features have been widely adopted in rehabilitation and movement science as indicators of neuromuscular engagement.

Recent studies show that machine learning models, especially XGBoost (eXtreme Gradient Boosting), can help model muscle behavior and support clinical decisions [20,21,22]. For example, advanced deep learning models have accurately identified rotator cuff tears from X-rays, showing how artificial intelligence (AI) can improve screening and diagnosis [23]. Overall, inertial measurement unit (IMU) data combined with XGBoost, has also been used to assess patient recovery status after rotator cuff surgery by providing objective measures of rehabilitation progress [12] as well as outpatient cases [22].

XGBoost is a powerful ensemble learning algorithm based on gradient-boosted decision trees. It has shown promise in different kinds of medical domains. According to Huang et al. [24] and Wang et al. [25], their model was able to outperform logistic regression in predicting outcomes such as hemodialysis-related blood pressure and mortality in traumatic brain injury patients. Similarly, Inoue et al. [26] used XGBoost to analyze spinal cord injury data with strong results. Kim [27] applied it to knee pain by combining physical and mental health factors showing that XGB can be used where patient data varies.

Researchers have used XGBoost to predict joint movement from electromyography (EMG) signals. Lu et al. [28] was able to achieve accurate joint angle predictions using simple EMG data, while Liu et al. [29] showed that biases might affect walking patterns and highlighted the need for adaptive models. Garcia et al. [30] stressed the importance of standard EMG recording methods for reliable and clinically useful machine learning. However, most studies are not run in real time and mainly use data from healthy subjects, which may be a limitation in real clinical settings.

Related research in EMG-based gesture recognition and rehabilitation robotics further demonstrates the applicability of machine learning to physiological signal interpretation. Rani et al. [31] reviewed over 150 studies and discovered trends such as combining different sensors and using personalized deep learning. Wang et al. [32] prioritized the need for energy efficient and real-time EMG systems for rehabilitation. These studies support the goal of building smart and responsive tools for shoulder recovery training.

XGBoost has also been successful in helping doctors assess clinical risk and make decisions. Zhang et al. [33] used it to predict which trauma patients would need treatment for bleeding, using shapley additive exPlanations (SHAP) to explain the results. Barnwal et al. [34] used it to estimate recovery times, showing how it can support patient care. Anatomical and imaging studies might also help improve model accuracy by connecting body structure with function. According to the research, Seo et al. [35] used MRI to study rotator cuff tears and mapped them to tendon anatomy which was useful for choosing model features. Kim [27] showed that imaging alone does not fully explain pain, which explains that it is important to include physical, mental, and social factors.

Building on these insights, Alaiti et al. [36] used machine learning to find patients less likely to recover well after rotator cuff surgery. This highlights how predictive models can help tailor rehabilitation technique to each patient. Combined models such as Random Forest, AdaBoost, and XGBoost have been compared in the past and in this research. Outside of healthcare, they have been used to predict complex, changing data such as in space weather forecasting [37]. Other studies have looked at how these models handle difficult tasks like working with unbalanced data and medical images. Rahman et al. [38] and Deshmukh and Bhosle [39] tested AdaBoost alongside other models like K-nearest neighbors (KNN), SVM and logistic regression, even with noisy or limited data. Azmi and Baliga [40] summed up this research and explained how boosting models (XGBoost and AdaBoost) manage the balance between bias and variance as a strong clinical tool.

Our study builds on these methods by combining mainly two fronts: predicting muscle activity using EMG data; and optimizing how rehabilitation time is spent during each session. Unlike past studies that focused on safe exercise levels [13], X-ray diagnosis [23], progress tracking [12], or predicting tear risks [22]; this study creates a personalized time plan for key shoulder movements within the typical allotted time for a PT session. The goal is to target surface-level muscles while reducing strain on deeper ones as sEMG signals are more reliable for superficial muscles.

The present research focuses on optimizing the allocation of time across four commonly prescribed arm movements within a 60 min therapy session: scaption, external rotation, internal rotation, and external rotation at 90° abduction. These exercises are frequently used in clinical settings to engage both superficial and deep muscles [41,42]. As a continuation of a previous study [43], we aim to build on existing knowledge and enhance shoulder rehabilitation efficiency by maximizing superficial muscle activity. Meanwhile, minimizing undue strain on deep stabilizers during early recovery stages should be maintained [44,45]. Accurate modeling of muscle activation patterns can improve treatment design and reduce reinjury risk [46,47]. Our study proposes a unique approach based on combination of powerful prediction models with a practical tool that helps plan exercise time. Instead of offering general advice or single diagnoses, this approach gives physical therapists a clear, data-backed logic to personalize rotator cuff rehabilitation for each patient.

## 2. Methodology

**Figure 1:**
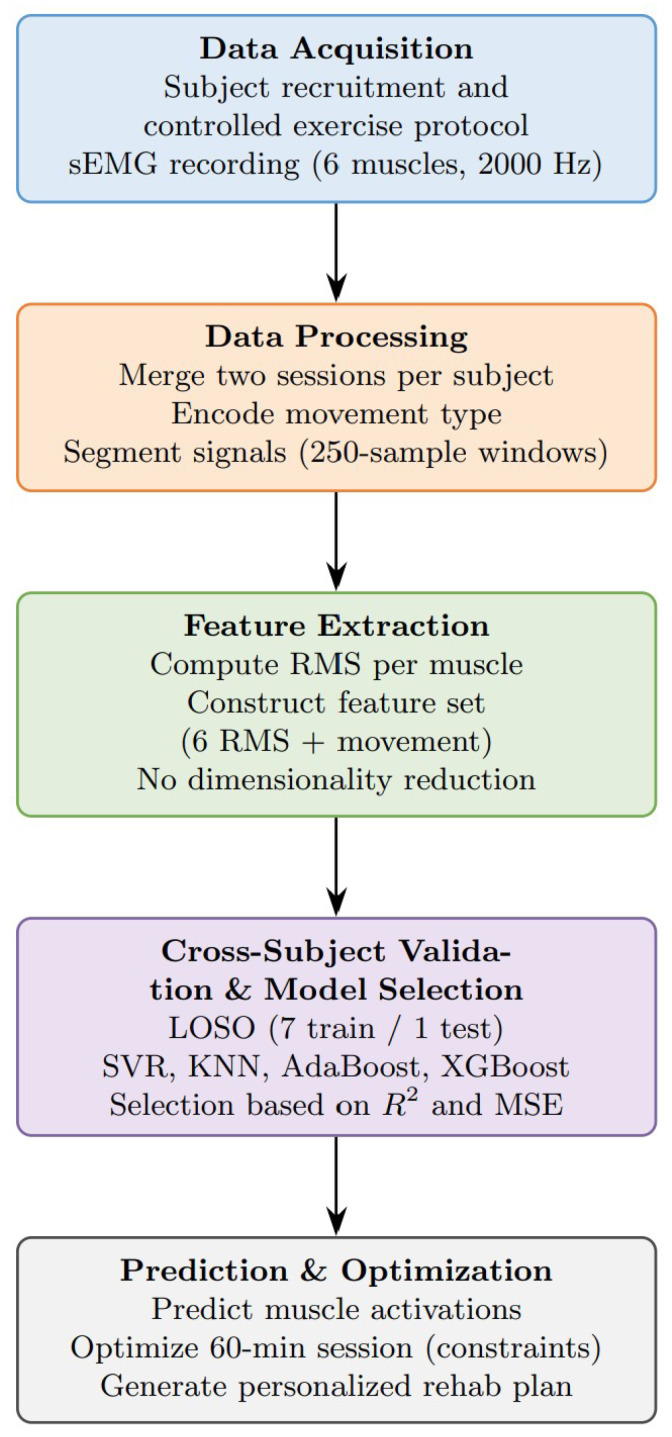
Data processing flowchart.

The structure of the paper is laid out in the flowchart below in Figure 1:

### 2.1. Data Collection

**Figure 2:**
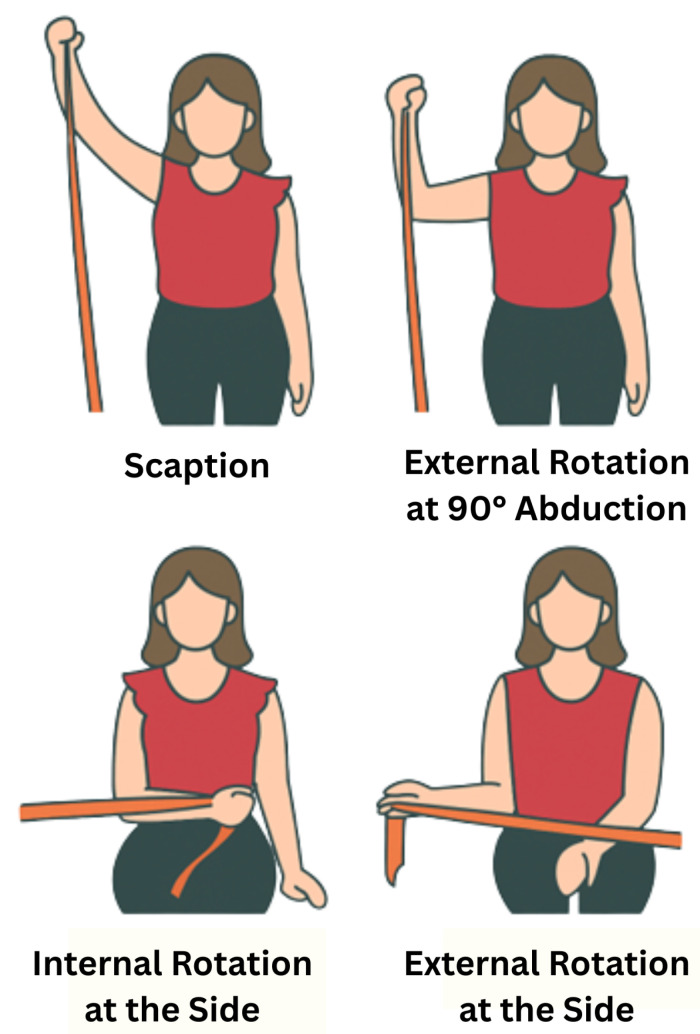
Illustration of the four rehabilitation exercises used for data collection.

For this study, the right shoulders of eight healthy individuals were assessed while performing four muscle exercises: scaption, internal rotation at the side, external rotation at the side, and external rotation at 90° abduction, as shown in Figure 2. Two datasets were collected for each subject to reduce potential biases. The subjects had a mean age of 20.2 ± 0.6 years (mean ± SD), and all of them had similar heights and anthropometric characteristics. Before starting the test, each subject filled out a consent form and a basic information form (see Institutional Review Board Statement). From the information sheet, it was found that all subjects were right-handed, which provides more consistency in the results between different subjects [43].

All exercises were performed under controlled kinematic conditions. Movement velocity, movement range, and execution pattern were standardized across participants through verbal instructions and visual demonstration. Subjects were instructed to avoid explosive movements and ballistic motion in order to minimize stretch-shortening cycle effects. This controlled environment was selected to isolate neuromuscular activation patterns and reduce biomechanical confounding factors during model development.

EMG signals were recorded using surface electromyography (sEMG) sensors placed on six muscles critical to rotator cuff rehabilitation. These included three superficial muscles (medial deltoid, posterior deltoid, trapezius) and three deeper muscles (supraspinatus, infraspinatus, teres minor). The signals were collected as time-series recordings of muscle activation during each exercise. Table 1 summarizes the primary muscles activated during each exercise, classified by anatomical layer.

sEMG provides a non-invasive and practical method for real-time monitoring, but it is more accurate for superficial muscles. Its ability to measure deep muscle activity is limited due to signal cross-talk from adjacent muscles. In contrast, needle EMG requires inserting electrodes into the muscle which makes it invasive. Although needle EMG offers greater specificity, its invasiveness makes it impractical for dynamic rehabilitation tasks. Therefore, sEMG was selected for its clinical feasibility and ease of use.

sEMG acquisition followed established methodological standards. MVC (Maximum Voluntary Contraction) normalization was not performed in this pilot study; however, isometric MVC is widely accepted as the reference standard for controlling inter-individual variability [48]. Instead, *Peak Normalization* has been performed to interpret results in a more consistent and meaningful fashion. Electrode placement adhered to anatomical best practices, with SENIAM (Surface EMG for Non-Invasive Assessment of Muscles) guidelines used to improve signal reliability and minimize inter-muscle signal contamination [49]. RMS feature extraction and temporal windowing were applied during preprocessing. Explicit artifact detection (e.g., motion noise and electrode drift) was not implemented in this work. sEMG signals were acquired at a sampling frequency of 2000 Hz using a NORAXON system (Scottsdale, AZ, USA). Hardware band-pass filtering (20–450 Hz) and a notch filter at 60 Hz were applied during acquisition. No additional digital filtering or post-hoc artifact removal was performed in this pilot study.

### 2.2. Sample Size Justification

To address concerns about the small number of samples, the study incorporated careful signal preprocessing, followed the same labeling process for all samples, and tested the models thoroughly. These steps helped reduce variability, minimize signal noise, and made sure the machine learning models could work well for different people. As a pilot investigation, feasibility of using machine learning to personalize PT was explored. We leveraged the small sample size to allow close monitoring of each session, ensuring high-quality signal capture, and carefully evaluating the performance of our modeling pipeline. At this stage, the goal was to test whether the proposed approach is technically viable and worth scaling to a larger, more diverse population. Similar sample sizes have been used in early-stage EMG and neuromechanics research, particularly when developing or validating new analytical methods. We believe this focused approach provides a meaningful first step toward integrating adaptive therapy models into rehabilitation practice.

### 2.3. Data Preprocessing and Feature Extraction

The raw sEMG data was preprocessed by merging both datasets for each subject to better train the model. A new column named “Movement” was added to each subject’s dataset to represent the categorical exercise type and was numerically encoded using label encoding to allow inclusion as a model feature; however, it was treated as contextual task information rather than as a surrogate for muscle activation. The intent was not for the model to classify exercise type, but to model how muscle activation patterns differ across functional tasks.

Root Mean Square (RMS) values were computed for each muscle to summarize the activation signals. This was done using a sliding window approach, where each window had 250 samples. The RMS value for each window was then computed.

In this pilot study, automated feature selection or dimensionality reduction was not applied. All six muscle RMS features and the encoded movement indicator were retained as model inputs. This decision was intentional because the feature set is small and each variable has a clear physiological meaning related to clinically relevant muscle activation in rotator cuff rehabilitation. Unlike high-dimensional sensing or imaging applications where feature redundancy is common, the present feature space is compact and reduces the risk of overfitting. Retaining all muscle features also allowed the model to capture inter-muscle coordination across subjects during cross-subject validation. As this framework is extended to larger datasets, future work will examine feature importance and selection methods such as SHapley Additive exPlanations (SHAP).

Following preprocessing, EMG signals were segmented into multiple RMS windows per exercise across two recording sessions for each subject, increasing the effective number of training samples while preserving subject-level separation during validation. Importantly, model performance was evaluated on held-out subjects rather than individual windows, ensuring that no data from the test subject contributed to training and reducing overfitting risk despite the limited cohort size.

### 2.4. Machine Learning Model Selection and Cross-Subject Validation

To determine the most suitable model for predicting muscle activation, several regression algorithms were compared, including Support Vector Regression (SVR), K-Nearest Neighbors (KNN), AdaBoost Regressor, and XGBoost.

Model selection followed a predefined criterion established prior to result analysis. Because the primary objective was to capture physiologically meaningful patterns in muscle activation, greater emphasis was placed on the coefficient of determination ($R^{2}$), reflecting how well each model explained variance in EMG signals across subjects. Mean Squared Error (MSE) was considered a secondary indicator of numerical precision.

For clinical translation, the ability to learn stable, generalizable activation patterns was prioritized over minimizing pointwise error. Accordingly, XGBoost was selected due to its superior $R^{2}$ performance, even though AdaBoost achieved lower MSE. This reflects a deliberate bias variance trade-off favoring consistency in physiological modeling over absolute error minimization.

These models were chosen because they strike a good balance between accuracy, interpretability, and responsiveness factors when working with a small dataset. More complex models like Random Forests and Neural Networks were excluded since they require more computing power and are more likely to overfit, especially with limited data. With just eight subjects, simpler models not only reduce the risk of overfitting but also make the approach more practical for real-world clinical use, where quick predictions and low computational cost really matter.

These models were tested using cross-subject validation to assess their accuracy and how well they could generalize. Instead of using a random train-test split, Leave-One-Subject-Out Cross-Validation was implemented to ensure robust generalization across subjects. For each iteration, data from seven subjects was used for training, and the remaining one subject’s data was used for testing. This method simulates real-world conditions where the model must generalize to unseen patients. The RMS values served as the input features.

XGBoost builds a sequence of simple decision trees, with each tree learning from the mistakes of the previous one. This way, it not only achieves strong predictive performance but also controls overfitting. XGBRegressor was chosen for this study because it effectively captures non-linear interactions between muscle activations and exercise movements without compromising computational efficiency.

XGBoost builds an additive model in a forward stage-wise manner; it allows optimization of an arbitrary differentiable loss function. At each step *t*, XGBoost adds a new function $f_{t}\left(x\right)$ to minimize the following regularized objective $L^{\left(t\right)}$:

(1) $$L^{\left(t\right)}=\sum_{i=1}^{n}l\left(y_{i},{\hat{y}}_{i}^{\left(t-1\right)}+f_{t}\left(x_{i}\right)\right)+\Omega \left(f_{t}\right)$$

where

- *l* is a differentiable loss function (such as mean squared error for regression);
- ${\hat{y}}_{i}^{\left(t-1\right)}$ is the prediction at iteration t−1;
- $f_{t}$ is the function (a decision tree) added at iteration *t*;
- $\Omega (f_{t})$ is a regularization term to penalize model complexity, encouraging simpler trees.

The regularization term $\Omega (f_{t})$ is defined as:

(2) $$\Omega \left(f\right)=\gamma T+\frac{1}{2}\lambda \sum_{j=1}^{T}w_{j}^{2}$$

where

- *T* is the number of leaves in the tree;
- $w_{j}$ is the weight of leaf *j*;
- γ and λ are regularization parameters.

In this framework, the predicted muscle activation value ${\hat{y}}_{i}$ for each observation *i* is computed by summing the outputs of all decision trees up to iteration *t*. At each boosting round, a new function $f_{t}\left(x\right)$ is fitted to the residual errors, updating the prediction as:

(3) $${\hat{y}}_{i}^{\left(t\right)}={\hat{y}}_{i}^{\left(t-1\right)}+f_{t}\left(x_{i}\right)$$

This iterative process minimizes the objective function $L^{\left(t\right)}$ by reducing the prediction error step by step. After all trees are added, the final ${\hat{y}}_{i}$ is used to calculate the Mean Squared Error (MSE) and the coefficient of determination ($R^{2}$). For other methods, ${\hat{y}}_{i}$ is obtained differently: in Support Vector Regression (SVR), it is the output of the fitted hyperplane; in K-Nearest Neighbors (KNN), it is the mean of the target values of the *k* nearest neighbors; and in AdaBoost, it is the weighted sum of predictions from multiple weak learners, usually shallow decision trees [50]. All models, including SVR, KNN, and AdaBoost, were implemented and evaluated using scikit-learn’s standard library functions.

Model performance was evaluated using two common regression metrics: Mean Squared Error (MSE) and R-squared ($R^{2}$), measured across all test subjects. MSE measures the average of the squares of the errors between the predicted and true muscle activation values. Lower MSE values indicate more accurate predictions by penalizing large errors more heavily. It is defined as:

(4) $$\mathrm{MSE}=\frac{1}{n}\sum_{i=1}^{n}{\left(y_{i}-{\hat{y}}_{i}\right)}^{2}$$

where

- $y_{i}$ represents the true activation value;
- ${\hat{y}}_{i}$ is the predicted value;
- *n* is the total number of samples.

The $R^{2}$ score quantifies the proportion of variance in the dependent variable that is predictable from the independent variables. It is calculated as:

(5) $$R^{2}=1-\frac{\sum_{i=1}^{n}{\left(y_{i}-{\hat{y}}_{i}\right)}^{2}}{\sum_{i=1}^{n}{\left(y_{i}-\bar{y}\right)}^{2}}$$

where $\bar{y}$ is the mean of the observed data.

An $R^{2}$ value closer to 1 indicates a better fit between the model predictions and the actual outcomes, while a negative $R^{2}$ suggests the model performs worse than simply predicting the mean of the data.

Using both metrics (MSE and $R^{2}$) provides a balanced and rigorous evaluation of model quality. While MSE assesses the magnitude of prediction errors, $R^{2}$ captures how well the model explains variance in the data. This dual-metric approach ensures that the selected model is both numerically precise and generalizable and critical qualities for clinical applications where both accuracy and interpretability are essential. This evaluation strategy is widely recommended in regression modeling practices.

### 2.5. Time Allocation Optimization

After predicting muscle activations, the next step was to optimize how the 60 min rehabilitation session should be divided among the different exercises. This duration was selected based on common clinical practice guidelines for outpatient PT, balancing sufficient therapeutic intensity with patient tolerance and scheduling feasibility. sEMG sensors primarily capture signals from superficial muscles more reliably than deep muscles; the goal was to increase the activation of superficial muscles compared to deep muscles, while keeping the time distribution balanced and practical for real-world use. The objective function was defined as:

(6) $$\mathrm{Objective}=-\left(w_{s}\sum_{i=1}^{n}x_{i}s_{i}-w_{d}\sum_{i=1}^{n}x_{i}d_{i}-0.1\cdot \mathrm{Var}\left(x\right)\right)$$

where

- *n* corresponds to the four rehabilitation exercises evaluated: scaption, internal rotation at the side, external rotation at the side, and external rotation at 90° abduction;
- $x_{i}$ represents the time allocated to exercise *i*;
- $s_{i}$ and $d_{i}$ represent the predicted superficial and deep muscle activations for each exercise *i*, calculated as the average predicted RMS values of the three superficial muscles (medial deltoid, posterior deltoid, trapezius) and the three deep muscles (supraspinatus, infraspinatus, teres minor), respectively;
- Var(x) penalizes high variance in time distribution across exercises;
- $w_{s}$ and $w_{d}$ are weights to control the emphasis on superficial versus deep muscles depending on rehabilitation goals.

Three different weighting strategies were evaluated:

- Case 1: 70% superficial/30% deep;
- Case 2: 50% superficial/50% deep;
- Case 3: 30% superficial/70% deep.

To address the challenge of optimally distributing exercise time, a constrained nonlinear optimization approach was implemented using the trust-constr algorithm from Python’s scipy.optimize.minimize function. This derivative-based method combines interior-point trust region techniques with Sequential Quadratic Programming (SQP), making it a good fit for problems with both equality constraints and bounds. The goal was to maximize the weighted sum of predicted superficial muscle activations, while also minimizing deep muscle strain and reducing excessive variability in time allocation across exercises.

To ensure that the resulting plans were clinically practical and aligned with real-world rehabilitation protocols, two main constraints were applied. First, an equality constraint ensured that the total exercise time always added up to exactly 60 min. Second, bound constraints kept each individual exercise within a practical range of 5 to 30 min. The superficial-to-deep weighting coefficients were selected to represent clinically plausible priority scenarios rather than fixed physiological constants. Specifically, the 70/30, 50/50, and 30/70 configurations were chosen to simulate distinct rehabilitation emphases ranging from superficial-dominant to relatively greater deep-muscle consideration. These values allowed examination of how time allocation responds to varying therapeutic goals.

The penalty was introduced to discourage extreme concentration of time in a single exercise and to promote clinically practical distribution across movements. The penalty magnitude was selected conservatively to influence allocation smoothness without overpowering the primary activation objective.

Bound constraints (5–30 min per exercise) were defined based on typical outpatient physical therapy session structures, ensuring that optimized recommendations remain clinically feasible and avoid unrealistic scheduling. The optimization process was initialized by assigning equal time to each exercise, and the solver consistently generated stable and interpretable time allocations under various superficial-to-deep muscle weighting scenarios. This enabled the study to test model robustness by examining how muscle group emphasis affected the optimized plans, supporting flexible, goal-driven rehabilitation strategies.

Additionally, the training time for each model was monitored using Python’s time module. The time was measured taking timestamps just before and after the training for each cross-validation fold. This helped to ensure which model was more efficient than the rest.

## 3. Results

### 3.1. Model Comparison and Selection

Table 2 summarizes the cross-subject validation results used to evaluate how well different regression models predict muscle activation from EMG features. All listed models accounted for seven muscles excluding the target muscle to train the predictive model considering the peak-normalized values. This approach eliminates the risk of trivial solutions.

To further assess model stability, fold-wise performance variability was examined across the eight Leave-One-Subject-Out folds. XGBoost demonstrated consistent performance across subjects, with limited dispersion in *R*^2^ and MSE values, indicating stable generalization. Reporting fold-level variability provides additional transparency regarding uncertainty and supports reproducibility of the evaluation protocol.

#### 3.1.1. Performance

Among all tested models, XGBoost demonstrated the most reliable and balanced predictive performance with the least average error. It achieved the highest $R^{2}$ value (0.5325), indicating that the model explains more than 53% of the variance in muscle activation levels across different subjects. To show cross-subject generalization, $R^{2}$ = 0.53 (Pearson Correlation factor **r = 0.73**) is substantial in neuromuscular modeling and indeed strong because sEMG datasets are extremely nonlinear, with high inter-trial variability and electrode placement sensitivity. In other words, 53% reflects variance in the target muscle RMS that is explained by the other muscles, which is actually a good prediction.

While AdaBoost produced the highest average MSE (0.0131 $\mu V^{2}$), its $R^{2}$ value (0.2230) was the lowest, suggesting it captured less of the overall activation dynamics. Other models such as SVR (MSE: 0.0109 $\mu V^{2}$, $R^{2}$: 0.3680) and KNN (MSE: 0.0094 $\mu V^{2}$, $R^{2}$: 0.4746, second-highest) underperformed on both metrics. These results highlight XGBoost’s strong ability to generalize across subjects and capture complex relationships within the EMG feature space.

#### 3.1.2. Efficiency

In addition to predictive accuracy, training time was also considered as a practical performance factor. XGBoost stood out as the second-fastest, completing the full cross-subject evaluation in just 43 s. KNN returned the fastest results at 25 s, though its predictive performance was significantly weaker. Despite achieving a slightly higher MSE, AdaBoost took much longer—480 s—to complete the same process with a significantly low average $R^{2}$ value (0.2230) which makes it an unsuitable approach for this case study. KNN presented the most accurate solution, requiring only 25 s to train and evaluate. With that, KNN is proven to be promising for use in such cases. However, based on the defined criteria, these results further support XGBoost as the most efficient and effective choice for this application.

The combination of high accuracy and low computation time makes XGBoost particularly well-suited for scalable, real-time rehabilitation systems. As a result, it was selected as the primary model for all subsequent optimization analyses.

### 3.2. XGBoost Evaluation Across All Muscles

**Figure 3:**
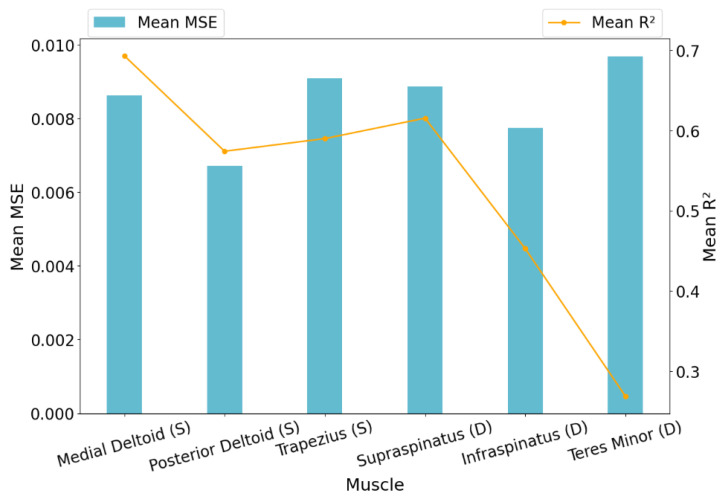
Mean MSE and $R^{2}$ of XGBoost for each muscle across all subjects based on cross-subject validation (grouped by anatomical layer: Superficial [S], Deep [D]).

Having been selected as the final model, XGBoost’s predictive performance was analyzed in more detail. It achieved an average MSE of 0.0084 $\mu V^{2}$ across all muscles and subjects, indicating strong overall accuracy. However, there was some variation in error across different muscles. The Teres Minor showed higher MSE values, suggesting more variability in its activation predictions, while muscles like the PostDeltoid and Infraspinatus were predicted with notably higher precision. Detailed results across individual muscles, based on cross-subject validation prior to optimization, are visualized in Figure 3. These figures provide important information; hence, more inferences from the results are noted in the Section 4.

### 3.3. Optimized Time Allocation

**Figure 4:**
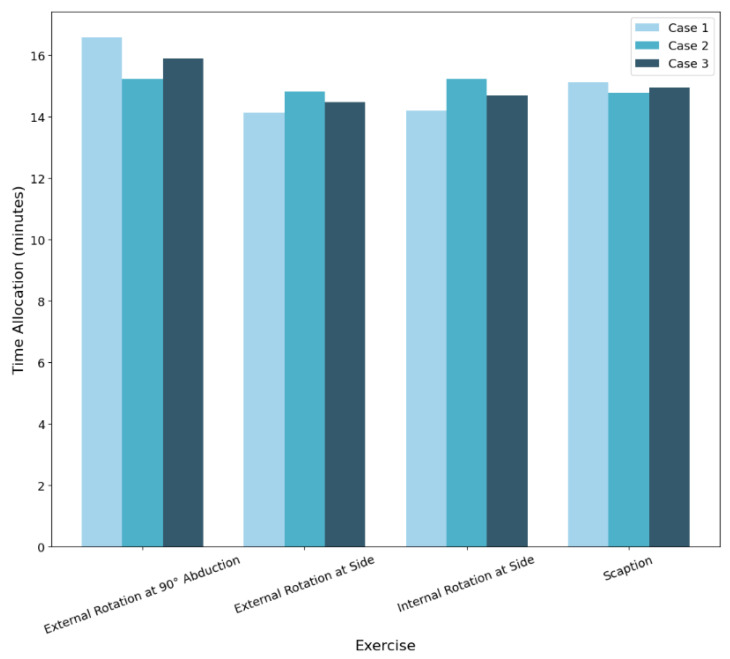
Optimized time allocation for all cases.

Following the prediction of muscle activation levels, an optimization framework was applied to determine the ideal time allocation for a 60-min rehabilitation session. The results of the optimized durations for each exercise under three different superficial-to-deep muscle weighting strategies are shown in Figure 4. For example, when the optimization emphasized reduced loading of deep muscles, external rotation at 90 deg abduction was assigned a greater portion of the session. Based on the present sEMG measurements, which primarily reflect superficial muscle activity (e.g., medial deltoid; Table 1), this movement was identified as a high-demand exercise that may help balance overall strain on deltoid and deep rotator cuff stabilizers.

#### 3.3.1. Case 1: 70% Superficial/30% Deep

In this case, the optimization prioritized the activation of superficial muscles. External rotation at 90° abduction (17 min) was allocated a significant portion of the session time followed by scaption (15 min), which contributed to both superficial and deep muscle engagement. Minimal time was assigned to internal and external rotation at the side (14 min each), reflecting their relatively lower superficial activation.

#### 3.3.2. Case 2: 50% Superficial/50% Deep

With equal weighting between superficial and deep muscles, the time allocation pattern remained consistent with Case 1. Internal rotation at side and external rotation at 90° abduction received the majority of the time (15 min each), indicating their balanced contribution to both muscle groups. This suggests that the model’s predictions are stable under moderate weighting changes.

#### 3.3.3. Case 3: 30% Superficial/70% Deep

When the priority was shifted towards deep muscle engagement, the time allocated to External Rotation at 90 deg abduction remained the predominant pick with about 16 min.

Overall, the relatively minor shifts in optimized time allocations between different weighting strategies indicate that the machine learning model predictions and optimization process were robust. This suggests that the system can reliably generate therapy session plans even when rehabilitation priorities (superficial versus deep muscle focus) are adjusted.

## 4. Discussion

This study demonstrated that machine learning models trained on sEMG data can support more efficient PT planning for rotator cuff recovery. sEMG signals were collected from eight participants and analyzed using an XGBoost Regressor which demonstrated robust predictive capabilities. The model achieved an average MSE of 0.0084 $\mu V^{2}$ and an *R*^2^ value of 0.5325 which reflected its effectiveness in capturing the variations of muscle activation during a variety of rehabilitation exercises. This work introduces a practical, data-driven framework that integrates EMG-based machine learning with an optimization strategy to support personalized rehabilitation exercise dosing. Rather than limiting the analysis to prediction or classification tasks, the proposed method converts estimated muscle activation patterns into clinically interpretable time-allocation recommendations. By combining cross-subject validation with constrained optimization, the framework is positioned as a decision-support approach that can assist individualized rehabilitation planning, rather than a rigid or prescriptive protocol.

To contextualize these findings within real-world clinical practice, it is important to clarify how the model outputs should be interpreted and applied. The optimized time allocations produced in this study should not be interpreted as fixed prescriptions, but rather as session-adaptive recommendations. The proposed framework is based on single-session data and does not account for neuromuscular adaptation across sessions. Future work will incorporate longitudinal, time-series modeling to enable adaptive dosage updates over the rehabilitation timeline.

Analysis of the sEMG dataset showed specific muscle activation patterns associated with specific movements. Through the model’s prediction, a 60 min rehabilitation session was designed using an optimization framework. The study noted that external rotation at 90° abduction consistently received the maximum allowable time allocation (>15 min) across all weighting strategies and highlighted its general efficiency. When the framework prioritized superficial muscle activity, scaption complemented abduction by receiving additional time allocation as a supportive movement, but it was not able to exceed abduction in significance.

Similarly, external rotation at 90deg abduction and scaption was chosen more often when the focus was on engaging deeper muscles. The small changes in time across different plans show that the model is both reliable and flexible and able to support different rehabilitation goals without losing consistency.

Importantly, the inclusion of varying superficial-to-deep weighting strategies was not intended to imply precise quantification of deep muscle loading, but rather to examine the stability of the optimization framework under different clinical priority assumptions. Across all three weighting scenarios (70/30, 50/50, and 30/70), the optimized time allocations remained largely consistent, with external rotation at 90° abduction consistently reaching the maximum allowable duration. The relatively minor variations observed across cases indicate that the optimization outcomes were primarily influenced by stable activation patterns learned by the predictive model, rather than by excessive sensitivity to weighting parameters. This consistency supports the robustness of the integrated machine learning and optimization framework and suggests that it can accommodate shifts in rehabilitation emphasis without producing unstable or clinically impractical session designs.

As illustrated in Figure 4, substantial shifts in superficial-to-deep weighting produced only moderate redistribution of session time while preserving the overall exercise ranking pattern. External rotation at 90° abduction consistently reached the upper time bound across all cases, and scaption exhibited controlled adjustments rather than abrupt changes. This stability under a 40% shift in weighting emphasis supports the robustness of the optimization framework and indicates that recommended dosage patterns are driven primarily by learned activation structure rather than by arbitrary parameter selection.

In addition to evaluating the primary model, a comparative analysis was conducted using other regression algorithms such as Support Vector Regression (SVR), K-Nearest Neighbors (KNN), and AdaBoost Regressor as results were listed in Table 2. XGB model outperformed all other methods by showing *R*^2^ = 0.53 and the minimum MSE values. Per-muscle RMS predictions included normalized RMS signals from all shoulder muscles except for the target per fold (excluding the target muscle itself) to capture comprehensive activation patterns during functional movements. This approach guarantees moderate accuracy (given EMG nonlinearity) but robust cross-subject generalization has been achieved (Table 2). These results supported the selection of XGBoost as the main modeling framework due to its strong predictive accuracy and ability to generalize across different subjects. It is worth noting that generalizability is supported by the use of peak-normalized values, as shown in Table 2. According to this Table, KNN and XGB completed the analysis in nearly the same amount of time, with KNN being slightly faster.

Additionally, Figure 3 provides a different insight into this investigation. The Trapezius (superficial) and Teres Minor (deep) both exhibited the highest reliability and the least variability in prediction across subjects.

In summary, this study shows that combining sEMG-based machine learning with optimization techniques is a promising and practical approach to support data-driven planning of physical therapy.

## 5. Limitations and Future Directions

Although the results are robust and encouraging, this pilot study was conducted on a small cohort of healthy, right-handed young adults under controlled conditions, which limits generalizability to clinical populations where pain and neuromuscular impairment alter activation patterns. Limited demographic diversity may also introduce bias. Future work will evaluate the framework in larger, clinically representative cohorts. The small sample size and the use of healthy, right-handed young adults restrict generalizability to clinical populations, where pain, compensatory strategies, and neuromuscular impairment alter muscle activation patterns. In addition, limited demographic diversity may introduce bias. Future work will incorporate MVC-based normalization, automated artifact detection, and calibration procedures to improve physiological reliability and enable inter-subject comparability.

Although two recording sessions per subject were merged to increase sample coverage, inter-session reliability was not formally assessed (e.g., via ICC). As a pilot study, validation relied on leave-one-subject-out cross-validation within a single dataset, and statistical reporting was limited to descriptive metrics without confidence intervals or hypothesis testing. Future work will incorporate formal reliability analysis and more rigorous statistical evaluation. The current framework focuses on time allocation, and future work may extend the model to incorporate additional dosage parameters relevant to clinical rehabilitation.

Surface electromyography (sEMG) is susceptible to signal crosstalk, particularly when estimating activation of deeper musculature in anatomically complex regions such as the shoulder. Although standardized electrode placement and RMS-based processing were applied, direct crosstalk quantification was not modeled. Therefore, deep-muscle activation in this study is treated as an indirect indicator, and future validation using needle EMG or imaging modalities would strengthen assessment.

The current framework also does not incorporate psychosocial factors such as pain perception, motivation, or treatment adherence, which are known to influence rehabilitation outcomes. Future work will integrate patient-reported and behavioral variables to support more comprehensive, person-centered modeling.

Despite strong predictive performance, additional steps are required for clinical deployment. Integration of explainable AI techniques such as SHAP will improve interpretability and clinician trust [51]. Furthermore, validation in patient populations and randomized controlled trials will be necessary to evaluate safety, effectiveness, and clinical impact. For practical implementation, future systems should integrate with wearable EMG devices, imaging modalities, and existing clinical software platforms to ensure usability and compatibility.

Collectively, these considerations highlight important avenues for future work while supporting the feasibility and potential clinical relevance of the proposed framework.

## 6. Conclusions

This study successfully demonstrated the efficiency of using a machine learning approach, specifically XGBoost, to optimize physical therapy dosage for rotator cuff rehabilitation based on sEMG data. The model achieved high predictive accuracy (*R*^2^ = 0.5325; MSE = 0.0084 $\mu V^{2}$), confirming its ability to predict muscle activation patterns across four commonly used shoulder movements. The cross-subject model achieved an average *R*^2^ of 0.53 (r = 0.73), indicating substantial inter-muscle coordination structure preserved across subjects despite physiological variability. Cross-subject sEMG prediction is extremely difficult because muscle geometry, electrode placement, skin impedance, and recruitment strategies vary, so explaining more than 50% of variance across individuals is clinically meaningful.

The optimization framework planned a 60 min therapy session, consistently giving the most time to external rotation at 90° abduction, followed by scaption, highlighting their importance in rehabilitation. The similar results across different muscle focus settings show that the system is reliable and that time allocations remain stable across various superficial-to-deep muscle weighting strategies. This framework demonstrates feasibility for modeling exercise time allocation in healthy cohorts, with potential relevance to personalized rehabilitation pending clinical validation.

Alongside XGBoost, a range of other machine learning regression models, including K-Nearest Neighbors (KNN), Support Vector Regression (SVR), and AdaBoost, were analyzed for their predictive performance. Although AdaBoost yielded the highest Mean Squared Error (0.0131 $\mu V^{2}$), its *R*^2^ score (0.2230) lagged behind that of all other models. Both SVR and KNN performed lower, showing reduced *R*^2^ values (0.37 and 0.47, respectively) and experiencing higher computational demands or error rates. However, KNN remains the runner-up and exhibited acceptable results. These results highlight the importance of choosing the right model in clinical machine learning.

This research demonstrates that optimizing exercise dosage through EMG-guided machine learning models offers a promising approach to rotator cuff rehabilitation. Across eight subjects, the model performance confirms that EMG signals can predict muscle activation across multiple arm movements. An optimization framework was developed to allocate a fixed 60 min therapy session across key exercises, providing a quantitative method for distributing time among movements to achieve improved outcomes.

The key contribution of this work is the development of a data-driven framework that bridges EMG-based muscle activation modeling with constrained optimization to support personalized rehabilitation dosage planning in a real-world healthcare application. By translating predicted activation patterns into interpretable time-allocation recommendations and validating the approach across subjects, this study demonstrates the feasibility of using machine learning not only for prediction but also for actionable decision support in rehabilitation settings. However, all clinical applications remain hypothetical until validated through randomized controlled trials in rotator cuff pathology patients.

## Figures and Tables

**Table 1:** Primary muscles activated per exercise with anatomical classification (S: Superficial, D: Deep).

Exercise	Activated Muscles
Scaption	Medial Deltoid (S) Trapezius (S) Supraspinatus (D) Infraspinatus (D) Teres Minor (D)
External Rotation at the Side	Posterior Deltoid (S) Infraspinatus (D) Teres Minor (D)
Internal Rotation at the Side	Medial Deltoid (S)
External Rotation at 90° Abduction	Posterior Deltoid (S) Supraspinatus (D) Teres Minor (D)

**Table 2:** Cross-subject validation performance and training time for different regression models *excluding target muscle*—peak normalized.

Model	Average MSE ($\mu V^{2}$)	Average $R^{2}$	Training Time (s)
SVR	0.0109	0.3680	560
KNN	0.0094	0.4746	**25**
AdaBoost	0.0131	0.2230	480
XGBoost	**0.0084**	**0.5325**	43

## Data Availability

The data presented in this study are available on request from the corresponding author.
